# Disparities of health expenditure associated with the experience of admission in long-term care hospital among patients with colorectal cancer in South Korea: A generalized estimating equation

**DOI:** 10.1371/journal.pone.0296170

**Published:** 2023-12-21

**Authors:** Woo-Ri Lee, Noorhee Son, Ki-Bong Yoo, Kyu-Tae Han

**Affiliations:** 1 Department of Research and Analysis, National Health Insurance Service Ilsan Hospital, Goyang-si, Republic of Korea; 2 Division of Cancer Control & Policy, National Cancer Control Institute, National Cancer Center, Goyang-si, Republic of Korea; 3 Division of Health Administration, College of Software and Digital Healthcare Convergence, Yonsei University, Wonju, Republic of Korea; Tehran University of Medical Sciences, ISLAMIC REPUBLIC OF IRAN

## Abstract

With rising concerns about the functional role of long-term care hospitals in the Korean medical system, this study aimed to observe the experience of admission in the long-term care hospitals and their association with medical expenditures among patients with colorectal cancer, and to investigate disparities among vulnerable populations. Data were obtained from the National Health Insurance Senior Cohort Database in South Korea for the period 2008–2019. With 6,305 patients newly diagnosed with colorectal cancer between 2008 and 2015, we conducted a regression analysis using the Generalized Estimating Equation model with gamma distribution to investigate the association between health expenditure and the experience of long-term care hospitals. We also explored the interaction effect of disability or income, followed by subgroup analysis. Among patients who received care at long-term care hospitals, the health expenditure within one year and five years after the incidence of colorectal cancer was found to be higher than in those who did not receive such care. It was observed that the low-income and disabled groups experienced higher disparities in health expenditure. The rise in health expenditure highlights importance for functional improvement, aligning with these initial purpose of long-term care hospitals to address the growing healthcare needs of the elderly population and ensure efficient healthcare spending, of long-term care hospitals. To achieve this original intent, it is imperative for government initiatives to focus on reducing quality gaps in long-term care hospital services and addressing cost disparities among individuals with cancer, including those with disabilities or low-income.

## Introduction

Cancer has been the primary cause of death in Korea for decades (age-standardized death rate, 254.4 per 100,000 in 2019; percentage of all deaths, 27.5% in 2019), resulting in considerable physical, psychological, and social burdens [1–3]. The Korean government has been aware of the need for cancer control and its responsibility in attaining this and has implemented many policy interventions. With cancer policies, various aspects of cancer control, such as accessibility, cost, and quality, have improved, particularly cancer coverage in Korea. This is in accordance with the policy to reduce patient copayment for cancer care, which has been introduced step-by-step since 2005, resulting in a fourfold reduction in the burden on patients with cancer (copayment level 20%–30% of the total cost to 5% of the total cost). This dramatic change can lead to moral hazards in the healthcare area, such as the explosion of healthcare utilization and long-term care hospitals (LTCHs) or preference for large hospitals.

LTCHs were stipulated by the Medical Service Act in 1994. Per-diem payments for LTCHs have been applied since 2008 to control medical costs. Over the decades, these hospitals have grown in number (number of LTCHs: 690 in 2008 to 1,435 in 2022) [4]. However, there are concerns regarding the functional role of LTCHs in the Korean medical system. The current distinction between LTCHs and facilities is ambiguous; older patients who need care but do not need treatment are admitted to LTCHs, leading to long-term hospitalization [5]. In terms of medical providers, LTCHs have fewer economic incentives to provide high-quality medical services due to per-diem payment methods; rather, the problem of risk selection has been pointed out [6].

Tertiary hospitals that mainly treat cancer in Korea do not admit patients other than during acute treatment for financial and institutional reasons. In this situation, patients frequently choose LTCHs as an alternative to isolation at home to help them recover from their condition. This is the reality of Korean LTCHs that are concerned about financial and moral hazards, because patients with cancer pay only 5% of the total medical cost, and they are relatively free to choose medical use [7]. Reduced spending stimulates another desire for consumption, prompting more medical utilization, such as high-quality or uninsured services [8]. In addition, many patients admitted to LTCHs are likely to be social admissions who have difficulty in rehabilitation and care, even if they return home after discharge, which leads to long-term hospitalization [9]. Consequently, medical expenditures for the use of LTCHs are exploding (Billion Korea Won, [% of the total medical cost], 998 [2.9%] in 2008 to 5,723 [6.0%] in 2021) [10].

Nevertheless, evidence and statistics related to the use of LTCHs among patients with cancer are insufficient in Korea, and there is a considerable need to lay the groundwork for institutional and policy improvement. Therefore, this study aimed to investigate the admission of LTCHs and its association with medical expenditure. Based on this study, we present the current status and basis for functional improvements in LTCHs in Korea.

## Materials and methods

### Study design and data

This study used data from the National Health Insurance Services (NHIS)-Senior cohort between 2008 and 2019. The NHIS provides cohort data upon request after reviewing the Institutional Review Board (IRB) approval and study protocol. These cohort data include a range of socioeconomic factors of the participants, such as sex, age, income, area of residence, type of insurance, and health-related factors, including medical service history and health screening results. Additionally, the dataset includes information on medical institutions [11]. The NHIS-Senior cohort data used in the study were a random sample stratified by sex, age, income, and area of residence, which comprised approximately 8% of the Korean older adult population aged 60 years and above in 2008. From 2009 onwards, an additional 8% of older adults aged 60 years were randomly sampled and included in the dataset. Currently, the NHIS-Senior cohort data include information on approximately one million older adults [11]. This study was exempted from review by the Institutional Review Board of the NCC (NCC2023-0146).

**Fig 1:**
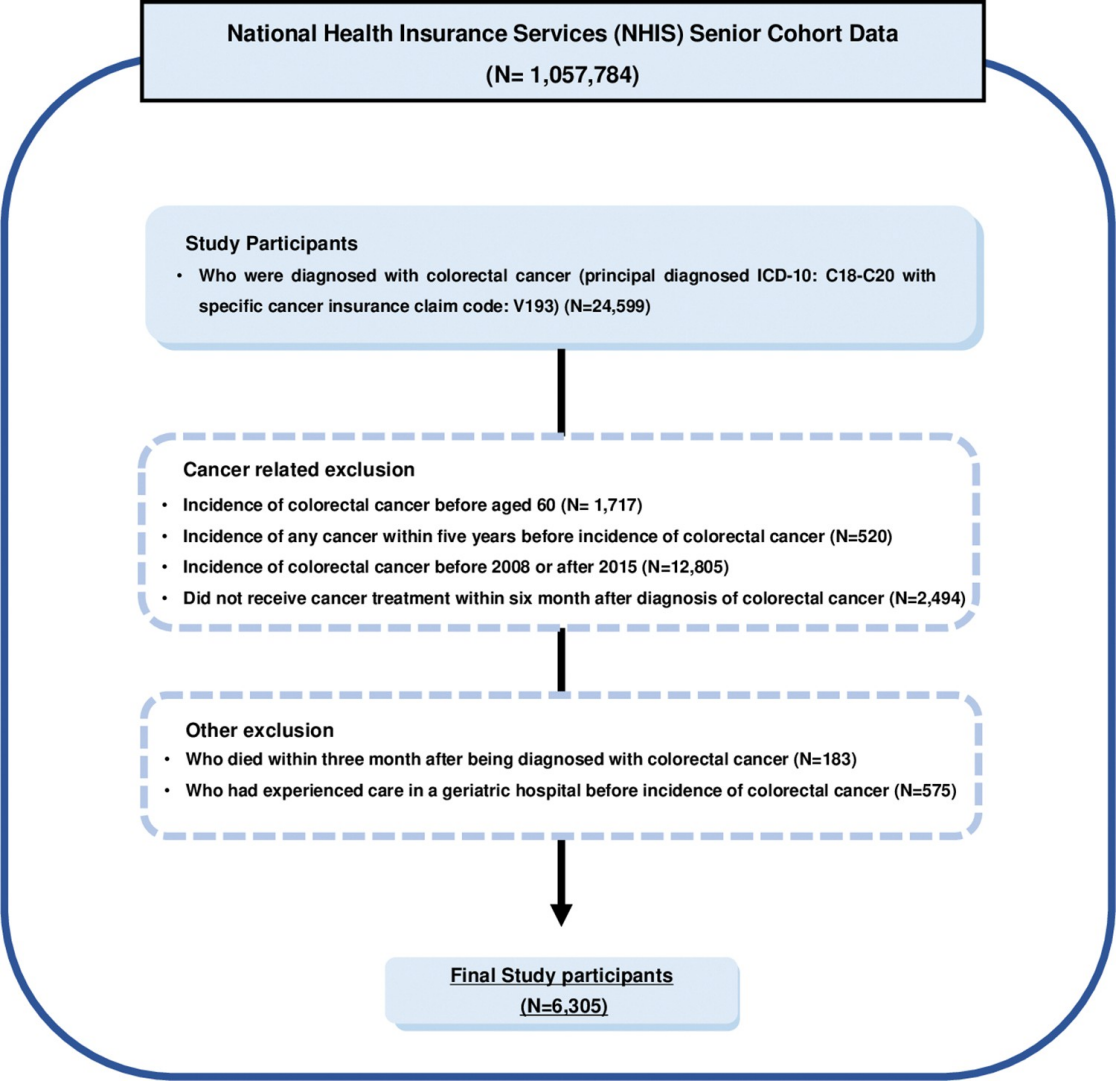
Selection process for the study population.

Of the 1,057,784 participants, those who were diagnosed with colorectal cancer (CRC) using the International Classification of Diseases, tenth version (ICD-10) code C18-C20 in the principal diagnosis and had claims with cancer-specific insurance claim code V193 were included as study participants (N = 24,599). Of these, participants who had CRC before the age of 60 years (N = 1,717), had a history of any cancer within 5 years before the incidence of CRC (N = 520), had an incidence of CRC before 2008 or after 2015 (N = 12,805), and had not received cancer treatment within 6 months after the incidence of CRC (N = 2,494) were excluded from the study based on cancer-related exclusion criteria. Furthermore, participants who died within 3 months after the incidence of CRC (N = 183) and those who had received healthcare at a LTCH before the incidence of CRC (N = 575) were also excluded. The final study included 6,305 participants (Fig 1).

### Variables

#### Outcome measure

The main outcome measure in our study was health expenditure, which we calculated in Korean won for the periods of one year and five years after the incidence of CRC. Total health expenditure was calculated by dividing the expenditure by the observation period (up to 365 days and up to 1,825 days). To account for annual inflation, health expenditure was analyzed using South Korea’s annual consumer prices, as reported by the World Bank. Given that the endpoint of our study is 2019, we established 2019 as the base year to reflect the inflation rate [12]. We considered the observation period, and the weights were multiplied by 365 days and 1,825 days depending on the observation period. The formula for calculating health expenditures is as follows:

One year health expenditure

= (sum of health expenditure (cost × annual inflation rate)) ÷ (sum of observation period (during diagnosis to 365 days)) × 365

Five year health expenditure

= (sum of health expenditure (cost × annual inflation rate)) ÷ (sum of observation period (during diagnosis to 1,825 days)) × 1,825

#### Independent variable

The primary independent variable was admission to long-term hospital care. Participants were assessed for their admission to LTCH within one year and five years after the incidence of CRC. Those admitted were further divided based on their length of stay (LOS) in LTCH, splitting them into below-median and above-median LOS groups. Consequently, the participants were classified into three categories: those without any LTCH admissions, those with below-median LOS during hospitalization, and those with above-median LOS.

#### Covariates

The covariates included socioeconomic and health-related factors. The socioeconomic factors included in this study were sex (male, female), age (60s, 70s, over 80s), income (below or above-median), region (Seoul, Gyeonggi, metropolitan, rural), and type of healthcare insurance (medical aid, NHI self-employed, NHI employees). Health-related factors included disability, Charlson Comorbidity Index (CCI), type of hospital, type of treatment, and death. Disability was categorized as nondisabled or disabled. The CCI scores were calculated using Quan’s criteria [13] and categorized as 0, 1, 2, or > 3. The type of hospital was classified as a tertiary, general, or other hospital based on the type of institution where the first CRC treatment was received. The type of treatment was categorized as surgery only, surgery and chemotherapy or radiotherapy, or chemotherapy or radiotherapy only based on the cancer treatment received. The occurrence of death was measured within one year and five years after the incidence of CRC. The year of CRC incidence was considered as another variable.

### Statistical analysis

T-test and ANOVA analyses were performed to investigate differences in health expenditures according to participants’ characteristics within one year and five years after the incidence of CRC. Statistical significance for the T-test and ANOVA was confirmed at p <0.05. Regression analysis was performed using a Generalized Estimating Equation (GEE) model with the log-link function to explore the relative risk (RR) of health expenditure depending on the admission in the long-term hospital care. GEE model with gamma distribution and log link is recognized for generating minimal biases and exhibits better performance in modelling positively skewed data, making it well-suited for the analysis of hospital cost data [14–16]. The results from gamma regression were interpreted based on the value of the relative risk after exponentiation of coefficient. In addition, we explored the interaction effect of disability or income, followed by subgroup analysis. Interaction effects of independent and stratified (disability, income) variables were confirmed, and statistical significance was confirmed at p<0.05, for type 3 analysis. All analyses were adjusted for covariates including sex, age, income, region, type of healthcare insurance, disability, CCI, type of hospital, type of treatment, death, and year of CRC incidence. Statistical significance tests for the regression analysis were considered significant when the lower bound of the 95% confidence interval (CI) did not include a 1. All analyses were performed using SAS version 9.4 (SAS Institute, Cary, NC, USA) for 2023.

### Ethical issues/statement

Informed consent was not required from the study participants and the requirement for ethical approval was waived by the Institutional Review Board of the National Cancer Center (NCC2023-0146) as a review exemption study.

## Results

### Patient characteristics

We compared the experience of long-term hospital care within one year and five years after CRC diagnosis based on the characteristics of the study participants (S1 Table). Females, older individuals, those with below-median income, residents in rural areas, those with medical aid, those with higher CCI scores, those treated in general hospitals, those who received only chemotherapy or radiotherapy, those who died, and those diagnosed with CRC in later years had a higher rate of long-term hospital care.

Health expenditures on participants’ characteristics within one year and five years after the incidence of CRC are shown in Table 1. Health expenditure is adjusted to account for the inflation rate based on the year 2019 Korean Won. The average health expenditures within one year and five years after the incidence of CRC were ₩9.5 million and ₩59.5 million, respectively. After comparing health expenditures between those admitted to long-term hospital care after the incidence of CRC and those who were not, we found that the average health expenditure of the admitted group was higher. For one year, the non-admitted group had an average health expenditure of ₩18.6 million, while the admitted group had ₩33.8 million for below-median LOS and ₩37.2 million for above-median LOS (p < .001). Over five years, the non-admitted group had an average health expenditure of ₩52.8 million, while the admitted group had ₩101.8 million for below-median LOS and ₩108.6 million for above-median LOS (p < .001).

**Table 1:** Health expenditure after the incidence of colorectal cancer.

Variable	Health Expenditure (Unit: 1,000,000 ₩)*					
	One year			Five year		
	N	Mean (±SD)	p-value	N	Mean (±SD)	p-value
**Total**	6305	19.5 (±16.7)		6305	59.5 (±83.4)	
**Admission of long-term care hospital**						
None	5982	18.6 (±15.9)	< .001	5508	52.8 (±79.7)	< .001
Admitted (below-median of LOS)	164	33.8 (±25.3)		406	101.8 (±101.0)	
Admitted (above-median of LOS)	159	37.2 (±18.6)		391	108.6 (±86.2)	
**Sex**						
Male	3908	19.8 (±16.9)	0.09	3908	59.6 (±83.6)	0.82
Female	2397	19.0 (±16.3)		2397	59.2 (±83.2)	
**Age**						
60s	3050	20.1 (±16.9)	0.007	3050	57.0 (±83.0)	0.04
70s	2794	19.1 (±16.5)		2794	61.1 (±83.1)	
over 80s	461	17.9 (±16.5)		461	65.7 (±88.3)	
**Income**						
Below-median	3542	19.8 (±16.5)	0.11	3542	60.8 (±83.2)	0.15
Above-median	2763	19.1 (±16.9)		2763	57.8 (±83.7)	
**Region**						
Seoul	1331	20.6 (±19.7)	0.03	1331	62.6 (±98.6)	0.18
Gyeonggi	1261	19.0 (±15.2)		1261	56.7 (±74.5)	
Metropolitan	1545	18.8 (±15.9)		1545	57.3 (±79.8)	
Rural	2168	19.6 (±16.1)		2168	60.7 (±80.7)	
**Type of healthcare insurance**						
Medical Aid	292	18.5 (±13.9)	0.33	292	63.0 (±71.1)	0.72
NHI Self-employed	1841	19.9 (±16.6)		1841	59.9 (±82.0)	
NHI Employee	4172	19.4 (±16.9)		4172	59.0 (±84.9)	
**Disability**						
Non-disabled	5353	19.2 (±16.1)	< .001	5353	57.4 (±80.1)	< .001
Disabled	952	21.3 (±19.5)		952	70.8 (±99.6)	
**CCI**						
0	1448	13.9 (±9.7)	< .001	1448	33.4 (±40.9)	< .001
1	893	15.1 (±10.4)		893	37.8 (±43.5)	
2	846	15.7 (±10.7)		846	41.0 (±47.6)	
≥3	3118	24.4 (±20.3)		3118	82.8 (±105.2)	
**Type of hospital**						
Tertiary hospital	3770	20.0 (±17.0)	< .001	3770	60.2 (±84.7)	< .001
General hospital	2126	19.6 (±15.6)		2126	61.2 (±79.5)	
Other	409	14.8 (±18.7)		409	43.9 (±90.0)	
**Type of treatment**						
Surgery only	3070	13.5 (±16.9)	< .001	3070	41.9 (±84.8)	< .001
Surgery & Chemo or radiotherapy	3028	24.7 (±14.0)		3028	71.3 (±74.5)	
Chemo or radiotherapy only	207	31.5 (±19.4)		207	145.9 (±101.6)	
**Death**						
Survivor	5868	17.3 (±11.6)	< .001	5868	28.3 (±22.2)	< .001
Died	437	48.9 (±35.9)		437	133.9 (±120.6)	
**Year of colorectal cancer incidence**						
2008	750	18.9 (±17.6)	0.02	750	55.2 (±84.0)	0.38
2009	852	20.2 (±16.2)		852	60.4 (±79.7)	
2010	863	20.1 (±16.4)		863	59.7 (±81.8)	
2011	919	20.3 (±18.8)		919	60.1 (±94.1)	
2012	967	18.7 (±14.7)		967	56.7 (±76.2)	
2013	1023	18.2 (±16.5)		1023	59.1 (±84.4)	
2014	931	20.1 (±16.6)		931	64.4 (±31.1)	

Note: CCI: Charlson comorbidity index; LOS: Length of stay; NHI: National Health Insurance

^*^Health expenditure is adjusted to account for the inflation rate based on the year 2019 Korean Won

### GEE analysis for health expenditure and the experience of long-term hospital care

We proceeded with a GEE analysis to investigate the association between health expenditure and the admission of long-term hospital care (Table 2). The RR of health expenditure within one year and five years after the incidence of CRC was higher for patients who were admitted to LTCH care compared to those who did not. Additionally, we observed that LOS was associated with higher average health expenditures. For one year, the admitted group had health expenditures 1.33 times higher for below-median LOS (95% CI 1.22–1.45) and 1.79 times higher for above-median LOS (95% CI 1.65–1.95) compared to the non-admitted group. Over five years, the admitted group had health expenditures 1.07 times higher for below-median LOS (95% CI 1.01–1.13) and 1.73 times higher for above-median LOS (95% CI 1.62–1.84) compared to the non-admitted group.

**Table 2:** Generalized estimating equations analysis on the association between long-term care hospital and health expenditure.

Variable	Health Expenditure			
	One year		Five year	
	RR	95% CI	RR	95% CI
**Admission of long-term care hospital**				
None	1.00		1.00	
Admitted (below-median of LOS)	1.33	(1.22–1.45)	1.07	(1.01–1.13)
Admitted (above-median of LOS)	1.79	(1.65–1.95)	1.73	(1.62–1.84)
**Sex**				
Male	1.00		1.00	
Female	0.97	(0.94–0.99)	1.00	(0.97–1.03)
**Age**				
60s	1.00		1.00	
70s	1.00	(0.97–1.02)	0.97	(0.94–0.99)
over 80s	0.95	(0.90–1.01)	0.90	(0.84–0.95)
**Income**				
Below-median	1.00		1.00	
Above-median	0.99	(0.96–1.01)	1.02	(0.99–1.05)
**Region**				
Seoul	1.00		1.00	
Gyeonggi	0.95	(0.91–0.99)	0.95	(0.91–0.99)
Metropolitan	0.96	(0.92–0.99)	0.99	(0.94–1.03)
Rural	0.95	(0.91–0.98)	0.96	(0.92–0.99)
**Type of healthcare insurance**				
Medical Aid	1.00		1.00	
NHI Self-employed	1.07	(0.99–1.14)	1.02	(0.95–1.10)
NHI Employee	1.05	(0.98–1.12)	1.01	(0.94–1.09)
**Disability**				
Non-disabled	1.00		1.00	
Disabled	1.11	(1.07–1.15)	1.15	(1.11–1.20)
**CCI**				
0	1.00		1.00	
1	1.07	(1.02–1.12)	1.11	(1.06–1.17)
2	1.11	(1.06–1.16)	1.11	(1.06–1.17)
≥3	1.42	(1.37–1.47)	1.45	(1.40–1.51)
**Type of hospital**				
Tertiary hospital	1.00		1.00	
General hospital	0.98	(0.95–1.01)	0.98	(0.95–1.02)
Other	0.79	(0.74–0.83)	0.84	(0.79–1.90)
**Type of treatment**				
Surgery only	1.00		1.00	
Surgery & Chemo or Radiotherapy	1.89	(1.83–1.94)	1.50	(1.45–1.54)
Chemo or Radiotherapy only	1.51	(1.39–1.63)	1.47	(1.35–1.61)
**Death**				
Survivor	1.00		1.00	
Died	2.52	(2.38–2.66)	3.92	(3.78–4.07)
**Year of colorectal cancer incidence**	1.00	(0.99–1.01)	1.02	(1.01–1.02)

A regression analysis using GEE model with gamma distribution and log-link function

Note: NHI: National Health Insurance; RR: Relative risk; CI: confidence interval; CCI: Charlson comorbidity index; LOS: Length of stay

### Subgroup analysis based on disability and income level

We proceeded to analyze the association between healthcare expenditure within specified groups based on disability and income levels. First, we analyzed the interaction term effects of stratified variables, disability and income, and experience of LTCH care using Type 3 analysis. The results confirmed the significant interaction effects of both disability and income with the admission in the LTCH (p < .001). The interaction effect analysis revealed that healthcare expenditure within one (Non-admitted*disabled: RR 1.11, 95% CI 1.07–1.15; below-median LOS*Non-disabled: RR 1.33, 95% CI 1.21–1.46; below-median LOS*Disabled: RR 1.46, 95% CI 1.14–1.88; above median LOS*Non-disabled RR 1.79, 1.64–1.97; above median LOS*Disabled RR 1.98, 95% CI 1.64–2.40) and five years (Non-admitted*disabled: RR 1.18, 95% CI 1.13–1.23; below-median LOS*Non-disabled: RR 1.10, 95% CI 1.03–1.18; below-median LOS*Disabled: RR 1.06, 95% CI 0.91–1.22; above median LOS*Non-disabled RR 1.77, 1.65–1.89; above median LOS*Disabled RR 1.82, 95% CI 1.58–2.10) were notably higher for disabled patients and those with increasing length of stay compared to non-disabled individuals without any experience of admission to LTCH (S2 Table). Furthermore, the analysis considering income levels indicates that healthcare expenditure within one (Non-admitted*Above median income: RR 0.99, 95% CI 0.96–1.02; below-median LOS*Below median income: RR 1.38, 95% CI 1.23–1.55; below-median LOS*Above median income: RR 1.24, 95% CI 1.08–1.42; above median LOS* Below median income RR 1.84, 1.65–2.05; above median LOS* Above median income: RR 1.72, 95% CI 1.51–1.95) and five years (Non-admitted*Above median income: RR 1.02, 95% CI 0.98–1.05; below-median LOS*Below median income: RR 1.05, 95% CI 0.97–1.13; below-median LOS*Above median income: RR 1.11, 95% CI 1.01–1.22; above median LOS* Below median income RR 1.75, 1.61–1.89; above median LOS* Above median income RR 1.73, 95% CI 1.56–1.91) were higher for low-income patients or those with increased length of stay compared to individuals with high income and no history of admission to LTCH (S3 Table).

**Fig 2:**
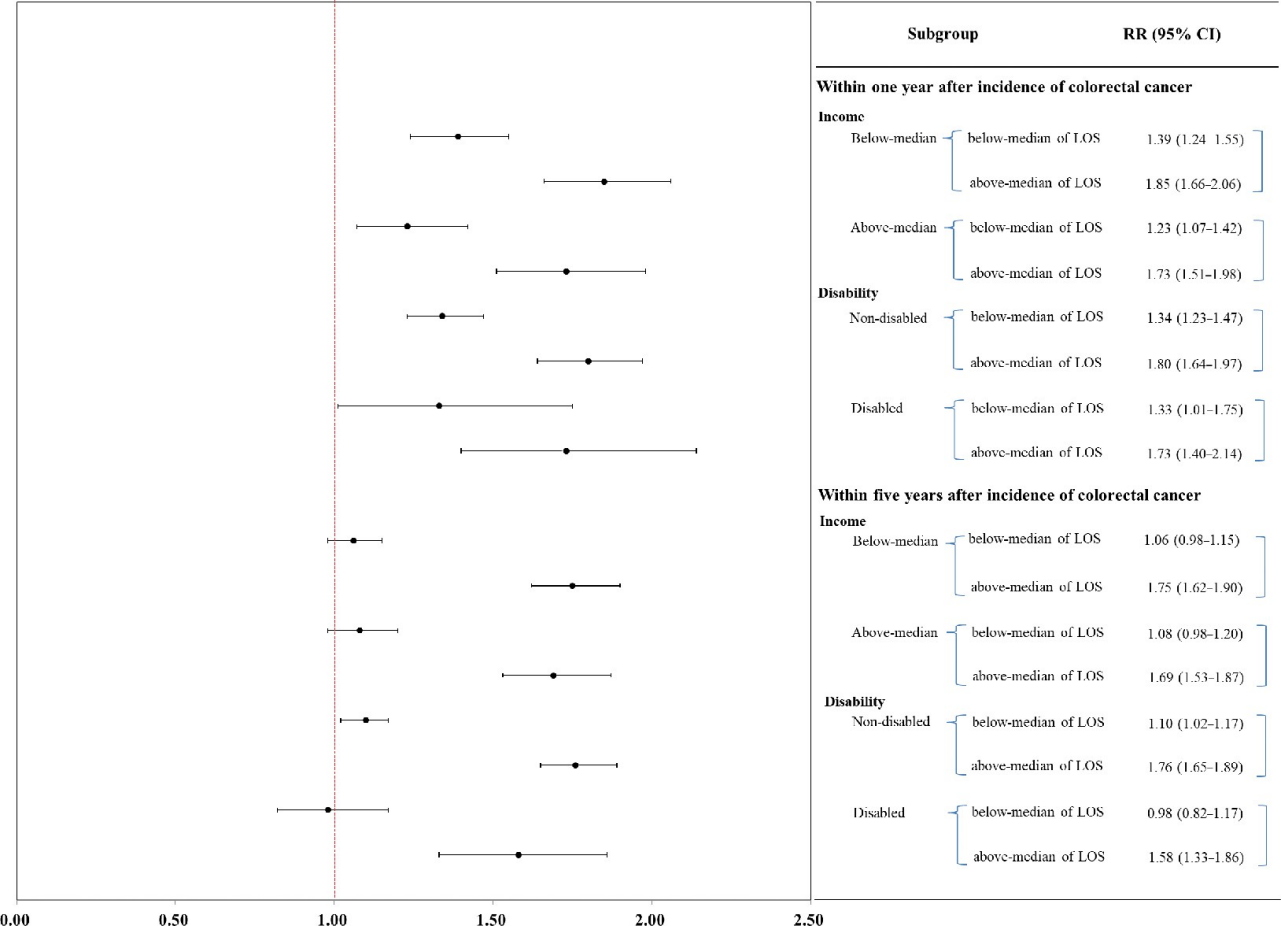
Results of subgroup analysis of GEE model.

Then, we conducted a subgroup analysis to investigate the health expenditures pattern according to income and disability (Fig 2). In the case of the non-disabled group, the health expenditure within one year after the incidence of CRC was 1.34 times higher for below-median LOS (95% CI 1.23–1.47) and 1.80 times higher for above-median LOS (95% CI 1.64–1.97) compared to the non-admitted group. And in the case of the disabled group, 1.33 times higher for below-median LOS (95% CI 1.01–1.75) and 1.73 times higher for above-median LOS (95% CI 1.40–2.14) compared to the non-admitted group. In the case of the non-disabled group, the health expenditure within five years after the incidence of CRC was 1.10 times higher for below-median LOS (95% CI 1.02–1.17) and 1.76 times higher for above-median LOS (95% CI 1.65–1.89) compared to the non-admitted group. In the case of the disabled group, no statistical significance of below-median LOS group (RR 0.98, 95% CI 0.82–1.17) but, 1.58 times higher for above-median LOS (95% CI 1.33–1.86) compared to the non-admitted group.

In the case of the below-median income group, the health expenditure within one year after the incidence of CRC was 1.39 times higher for below-median LOS (95% CI 1.24–1.55) and 1.85 times higher for above median income group (95% CI 1.66–2.06) compared to the non-admitted group. In the case of the above-median income group, 1.23 times higher for below-median LOS (95% CI 1.07–1.42) and 1.73 times higher for above-median LOS (95% CI 1.51–1.98) compared to the non-admitted group. In the case of the below-median income group, the health expenditure within five years after the incidence of CRC was not statistically significant of below median LOS group (RR 1.06, 95% CI 0.98–1.15) but, 1.75 times higher for above median LOS (95% CI 1.62–1.90) compared to the non-admitted group. and in the case of the disabled group, no statistical significance of below median LOS group (RR 1.08, 95% CI 0.98–1.20) but, 1.69 times higher for above median LOS (95% CI 1.53–1.87) compared to the non-admitted group.

## Discussion

To the best of our knowledge, this is the first study to investigate the relationship between the experience of LTCH and health expenses, and observe possible disparities in vulnerable populations. The study revealed that older patients who had utilized LTCHs had significantly higher medical expenses than those who did not. This result indicates that older patients with cancer utilize LTCHs to receive cancer care. Previous studies have also shown that individuals who utilize long-term care facilities for severe illnesses incur higher medical expenses than those who utilize other medical facilities [17]. Additionally, a study conducted in Korea reported an increasing trend in the utilization of LTCHs by patients with breast, colorectal, and gastric cancers between 2013 and 2017, accompanied by an increase in medical expenses [18]. In South Korea, LTCHs provide services for patients who require long-term healthcare, especially older patients [19]. Given the projected increase in healthcare utilization and expenditure for older adults, LTCHs play an important role in meeting the demand for services while promoting efficient healthcare spending [20]. However, concerns have been raised owing to the rapid increase in the number of LTCHs and the marketization of services.5 This competitive environment has led to excessive competition among LTCHs, resulting in unnecessary healthcare spending and increased health expenses [21, 22]. Considering the significant burden posed by cancer and the unnecessary healthcare spending pattern observed in LTCHs, the utilization of LTCHs among patients with cancer may have led to substantial medical expenses [23, 24].

The results of the interaction effect analysis based on disability and income status indicate that vulnerable patients are also subject to experiencing persistent physical and socioeconomic disparities when utilizing LTCH services as the health expenditure was higher for patients with disabilities and those in low-income groups compared to non-disabled individuals and high-income groups. These findings align with previous research indicating that patients with disabilities or low-income level incur higher expenses owing to the utilization of long-term care services. For disability, a study conducted in Japan found longer lengths of stay and higher healthcare expenses among disabled patients [25]. Similarly, a study conducted in the United States reported higher disease burden and higher long-term care expenses among disabled individuals than among the general older population [26]. In addition, a study conducted in Denmark estimated the healthcare expenditure for individuals with disabilities aged 55 years and above and projected a €16 billion increase in medical expenses due to disability-related illnesses by 2030, with two-thirds of the expenses attributable to long-term care facility usage [27]. The significant disparity in medical expenses among patients with cancer with disabilities using LTCHs underscores the importance of developing tailored healthcare and caregiving services for vulnerable patients with cancer. Patients with disabilities have poorer health status than those without disabilities [28]. The occurrence of diseases such as cancer leads to increased mortality rates and low quality of life, resulting in higher medical expenses compared to patients with cancer without disabilities [29, 30]. Therefore, proactive support should be provided to enable equitable access to medical and caregiving services for such patients [31]. However, the increased medical expenses among patients with cancer with disabilities indicates that vulnerable patients continue to experience healthcare inequality, particularly when utilizing LTCHs. For the subgroup analysis based on the income levels, patients with low-income level faced higher health expenditure over a five-year period when utilizing LTCH compared to the high-income patients. This aligns with previous studies indicating higher long-term care expenses in low-income groups [32–34]. It is important to note that patients with cancer, in general, are prone to financial difficulties due to the substantial burden of cancer [35]. The occurrence of cancer can increase the likelihood of experiencing financial hardships, which can result in non-adherence or insufficient cancer treatment [36]. Missing the appropriate treatment period for cancer can lead to higher healthcare expenses in the future, ultimately impacting patient outcomes and exacerbating disparities even further [37, 38].

The higher health expenditure associated with the experience of LTCHs underscore the importance of improving the functionality of LTCHs for patients with cancer, with a specific focus on individuals with disabilities and those belonging to low-income groups. Currently, LTCHs in South Korea continue to face controversies regarding overtreatment, social hospitalization, and increased prolonged stays, resulting in unnecessary expenditure [6, 39]. With the challenge of becoming a super-aged society by 2025, discussions regarding the establishment of integrated community care systems to effectively provide comprehensive support are ongoing [19]. In addition to these efforts, there is a need to develop tailored interventions specifically designed for patients with cancer. This involves improving the functionality of LTCHs to better accommodate the needs of patients with cancer and ensure that cancer treatment and care are provided to vulnerable populations without any disparities.

Nevertheless, this study had several limitations. First, owing to data limitations, this study was unable to consider the cancer stage at the time of diagnosis. To overcome this limitation, individuals who died within three months after cancer occurrence and those who did not receive treatment within six months after diagnosis were excluded. Furthermore, measuring health expenditure is limited, as it often fails to capture non-medical costs, such as caregiving expenses that may arise in LTCH services. Caregiving expenses incurred in LTCHs are significant costs that patients must bear entirely and are important determinants of the utilization of LTCHs among patients. Further research should be conducted to investigate the effects of caregiving expenses using additional surveys. Despite these limitations, to the best of our knowledge, this study is significant as it is the first to demonstrate differences in health expenditure based on the experience of LTCHs among patients with cancer in Korea. The findings of this study have significant policy implications, as they provide evidence related to the use of LTCH among patients with cancer and highlight persistent physical and socioeconomic disparities within vulnerable populations when it comes to providing LTCH services.

## Conclusion

In conclusion, our study revealed increased health expenditure risks among patients with cancer who have utilized LTCHs, along with significant disparities in health expenditures among patients with disabilities and those with low income. The observed patterns of excessive healthcare spending within LTCH underscore the necessity for functional improvement to effectively address increasing healthcare demands while managing healthcare costs. These findings emphasize the significance of government efforts aimed at enhancing service quality and controlling excessive healthcare spending within LTCH in South Korea.

## Supporting information

S1 Table General characteristics of the study population. Descriptive statistics and associations between the multiple covariates and experience of long-term care hospitals.
(DOCX)

S2 Table Results of the interaction effect between experience of long-term care hospital care and disability. Investigation of interaction term effects of disability and experience of long-term hospital care using Type 3 analysis.
(DOCX)

S3 Table Results of the interaction effect between experience of long-term care hospital care and income. Investigation of interaction term effects of income and experience of long-term hospital care using Type 3 analysis.
(DOCX)
