# Supplementary material for: Disparities of health expenditure associated with the experience of admission in long-term care hospital among patients with colorectal cancer in South Korea: A generalized estimating equation
Source: PLoS One. 2023 Dec 21;18(12):e0296170. doi: 10.1371/journal.pone.0296170 (PMC10735009; doi:10.1371/journal.pone.0296170)
Supplement: S1 Table — Descriptive statistics and associations between the multiple covariates and experience of long-term care hospitals. (DOCX) [file pone.0296170.s001.docx]

**Supplementary Table 1. General characteristics of the study population**

| **Variable** | **Admission of long-term care hospital** | | | | | | | | |
| --- | --- | --- | --- | --- | --- | --- | --- | --- | --- |
|  | **One year** | | | | **Five year** | | | | |
|  | **None** | **Admitted (below median of LOS)** | **Admitted (above median of LOS)** | **p-value** | **None** | **Admitted (below median of LOS)** | | **Admitted (above median of LOS)** | **p-value** |
|  | **N(%)** | **N(%)** | **N(%)** |  | **N(%)** | **N(%)** | | **N(%)** |  |
| **Total** | 5982 (94.9) | 164 (2.6) | 159 (2.5) |  | 5508 (87.4) | 406 (6.4) | | 391 (6.2) |  |
| **Sex** |  |  |  |  |  |  | |  |  |
| Male | 3745 (95.8) | 79 (2.0) | 84 (2.1) | <.001 | 3507 (89.7) | 224 (5.7) | | 177 (4.5) | <.001 |
| Female | 2237 (93.3) | 78 (3.3) | 82 (3.4) |  | 2001 (83.5) | 182 (7.6) | | 214 (8.9) |  |
| **Age** |  |  |  |  |  |  | |  |  |
| 60s | 2945 (96.6) | 49 (1.6) | 56 (1.8) | <.001 | 2814 (92.3) | 131 (4.3) | | 105 (3.4) | <.001 |
| 70s | 2634 (94.3) | 77 (2.8) | 83 (3.0) |  | 2354 (84.3) | 219 (7.8) | | 221 (7.9) |  |
| over 80s | 403 (87.4) | 31 (6.7) | 27 (5.9) |  | 340 (73.8) | 56 (12.1) | | 65 (14.1) |  |
| **Income** |  |  |  |  |  |  | |  |  |
| Below median | 3352 (94.6) | 92 (2.6) | 98 (2.8) | 0.19 | 3069 (86.6) | 239 (6.7) | | 234 (6.6) | 0.33 |
| Above median | 2630 (95.2) | 65 (2.4) | 68 (2.5) |  | 2439 (88.3) | 167 (6.0) | | 157 (5.7) |  |
| **Region** |  |  |  |  |  |  | |  |  |
| Seoul | 1275 (95.8) | 26 (2.0) | 30 (2.3) | 0.01 | 1187 (89.2) | 79 (5.9) | | 65 (4.9) | 0.01 |
| Gyeonggi | 1206 (95.6) | 31 (2.5) | 24 (1.9) |  | 1114 (88.3) | 86 (6.8) | | 61 (4.8) |  |
| Metropolitan | 1459 (94.4) | 44 (2.8) | 42 (2.7) |  | 1340 (86.7) | 100 (6.5) | | 105 (6.8) |  |
| Rural | 2042 (94.2) | 56 (2.6) | 70 (3.2) |  | 1867 (86.1) | 141 (6.5) | | 160 (7.4) |  |
| **Type of healthcare insurance** |  |  |  |  |  |  | |  |  |
| Medical Aid | 268 (91.8) | 14 (4.8) | 10 (3.4) | 0.06 | 229 (78.4) | 27 (9.2) | | 36 (12.3) | <.001 |
| NHI Self-employed | 1742 (94.6) | 46 (2.5) | 53 (2.9) |  | 1609 (87.4) | 116 (6.3) | | 116 (6.3) |  |
| NHI Employee | 3972 (95.2) | 97 (2.3) | 103 (2.5) |  | 3670 (88.0) | 263 (6.3) | | 239 (5.7) |  |
| **Disability** |  |  |  |  |  |  | |  |  |
| Non-disabled | 5079 (94.9) | 139 (2.6) | 135 (2.5) | 0.78 | 4693 (87.7) | 339 (6.3) | | 321 (6.0) | 0.1 |
| Disabled | 903 (94.9) | 18 (1.9) | 31 (3.3) |  | 815 (85.6) | 67 (7.0) | | 70 (7.4) |  |
| **CCI** |  |  |  |  |  |  | |  |  |
| 0 | 1411 (97.4) | 20 (1.4) | 17 (1.2) | <.001 | 1324 (91.4) | 68 (4.7) | | 56 (3.9) | <.001 |
| 1 | 875 (98.0) | 8 (0.9) | 10 (1.1) |  | 817 (91.5) | 32 (3.6) | | 44 (4.9) |  |
| 2 | 815 (96.3) | 12 (1.4) | 19 (2.2) |  | 758 (89.6) | 38 (4.5) | | 50 (5.9) |  |
| ≥3 | 2881 (92.4) | 117 (3.8) | 120 (3.8) |  | 2609 (83.7) | 268 (8.6) | | 241 (7.7) |  |
| **Type of hospital** |  |  |  |  |  |  | |  |  |
| Tertiary hospital | 3580 (95.0) | 92 (2.4) | 98 (2.6) | 0.33 | 3313 (87.9) | 229 (6.1) | | 228 (6.0) | 0.56 |
| General hospital | 2007 (94.4) | 56 (2.6) | 63 (3.0) |  | 1834 (86.3) | 147 (6.9) | | 145 (6.8) |  |
| Other | 395 (96.6) | 9 (2.2) | 5 (1.2) |  | 361 (88.3) | 30 (7.3) | | 18 (4.4) |  |
| **Type of treatment** |  |  |  |  |  |  | |  |  |
| Surgery only | 2920 (95.1) | 69 (2.2) | 81 (2.6) | <.001 | 2716 (88.5) | 160 (5.2) | | 194 (6.3) | <.001 |
| Surgery & Chemo or radiotherapy | 2885 (95.3) | 70 (2.3) | 73 (2.4) |  | 2633 (87.0) | 215 (7.1) | | 180 (5.9) |  |
| Chemo or radiotherapy only | 177 (85.5) | 18 (8.7) | 12 (5.8) |  | 159 (76.8) | 31 (15.0) | | 17 (8.2) |  |
| **Death** |  |  |  |  |  |  | |  |  |
| Survivor | 5651 (96.3) | 92 (1.6) | 125 (2.1) | <.001 | 4183 (94.1) | 100 (2.3) | | 161 (3.6) | <.001 |
| Died | 331 (75.7) | 65 (14.9) | 41 (9.4) |  | 1325 (71.2) | 306 (16.4) | | 230 (12.4) |  |
| **Year of colorectal cancer incidence** |  |  |  |  |  |  | |  |  |
| 2008 | 731 (97.5) | 10 (1.3) | 9 (1.2) | <.001 | 680 (90.7) | 37 (4.9) | | 33 (4.4) | <.001 |
| 2009 | 816 (95.8) | 21 (2.5) | 15 (1.8) |  | 756 (88.7) | 49 (5.8) | | 47 (5.5) |  |
| 2010 | 828 (95.9) | 13 (1.5) | 22 (2.5) |  | 778 (90.2) | 38 (4.4) | | 47 (5.4) |  |
| 2011 | 877 (95.4) | 24 (2.6) | 18 (2.0) |  | 802 (87.3) | 63 (6.9) | | 54 (5.9) |  |
| 2012 | 915 (94.6) | 22 (2.3) | 30 (3.1) |  | 845 (87.4) | 57 (5.9) | | 65 (6.7) |  |
| 2013 | 953 (93.2) | 31 (3.0) | 39 (3.8) |  | 867 (84.8) | 80 (7.8) | | 76 (7.4) |  |
| 2014 | 862 (92.6) | 36 (3.9) | 33 (3.5) |  | 780 (83.8) | 82 (8.8) | | 69 (7.4) |  |
| *Note: National Health Insurance; CCI: Charlson comorbidity index; LOS: Length of stay* | | | | | | |  | | |
