# Supplementary material for: Disparities of health expenditure associated with the experience of admission in long-term care hospital among patients with colorectal cancer in South Korea: A generalized estimating equation
Source: PLoS One. 2023 Dec 21;18(12):e0296170. doi: 10.1371/journal.pone.0296170 (PMC10735009; doi:10.1371/journal.pone.0296170)
Supplement: S2 Table — Investigation of interaction term effects of disability and experience of long-term hospital care using Type 3 analysis. (DOCX) [file pone.0296170.s002.docx]

**Supplementary Table 2. Results of the interaction effect between experience of long-term care hospital care and disability**

| **Variable** | **Health Expenditure** | | | | | |
| --- | --- | --- | --- | --- | --- | --- |
|  | **One year** | | | **Five year** | | |
|  | **RR** | **95% CI** | **Type 3 p-value** | **RR** | **95% CI** | **Type 3 p-value** |
| **Admission of long-term care hospital * Disability** |  |  |  |  |  |  |
| None * Non-disabled | 1.00 |  | <.001 | 1.00 |  | <.001 |
| None * Disabled | 1.11 | (1.07–1.15) |  | 1.18 | (1.13–1.23) |  |
| Admitted (below median of LOS) * Non-disabled | 1.33 | (1.21–1.46) |  | 1.10 | (1.03–1.18) |  |
| Admitted (below median of LOS) * Disabled | 1.46 | (1.14–1.88) |  | 1.06 | (0.91–1.22) |  |
| Admitted (above median of LOS) * Non-disabled | 1.79 | (1.64–1.97) |  | 1.77 | (1.65–1.89) |  |
| Admitted (above median of LOS) * Disabled | 1.98 | (1.64–2.40) |  | 1.82 | (1.58–2.10) |  |
| **Sex** |  |  |  |  |  |  |
| Male | 1.00 |  | 0.02 | 1.00 |  | 0.83 |
| Female | 0.97 | (0.94–0.99) |  | 1.00 | (0.97–1.03) |  |
| **Age** |  |  |  |  |  |  |
| 60s | 1.00 |  | 0.26 | 1.00 |  | 0.002 |
| 70s | 1.00 | (0.97–1.02) |  | 0.97 | (0.94–1.01) |  |
| over 80s | 0.95 | (0.90–1.01) |  | 0.90 | (0.84–0.96) |  |
| **Income** |  |  |  |  |  |  |
| Below median | 1.00 |  | 0.35 | 1.00 |  | 0.34 |
| Above median | 0.99 | (0.96–1.01) |  | 1.02 | (0.98–1.05) |  |
| **Region** |  |  |  |  |  |  |
| Seoul | 1.00 |  | 0.02 | 1.00 |  | 0.07 |
| Gyeonggi | 0.95 | (0.91–0.99) |  | 0.95 | (0.90–0.99) |  |
| Metropolitan | 0.96 | (0.92–0.99) |  | 0.99 | (0.94–1.03) |  |
| Rural | 0.95 | (0.91–0.98) |  | 0.96 | (0.92–0.99) |  |
| **Type of healthcare insurance** |  |  |  |  |  |  |
| Medical Aid | 1.00 |  | 0.14 | 1.00 |  | 0.71 |
| NHI Self-employed | 1.07 | (0.99–1.14) |  | 1.02 | (0.95–1.10) |  |
| NHI Employee | 1.05 | (0.98–1.12) |  | 1.01 | (0.94–1.09) |  |
| **CCI** |  |  |  |  |  |  |
| 0 | 1.00 |  | <.001 | 1.00 |  | <.001 |
| 1 | 1.07 | (1.02–1.12) |  | 1.11 | (1.06–1.17) |  |
| 2 | 1.11 | (1.06–1.16) |  | 1.11 | (1.06–1.17) |  |
| ≥3 | 1.42 | (1.37–1.47) |  | 1.45 | (1.40–1.51) |  |
| **Type of hospital** |  |  |  |  |  |  |
| Tertiary hospital | 1.00 |  | <.001 | 1.00 |  | <.001 |
| General hospital | 0.98 | (0.95–1.01) |  | 0.98 | (0.95–1.02) |  |
| Other | 0.79 | (0.74–0.83) |  | 0.84 | (0.79–0.90) |  |
| **Type of treatment** |  |  |  |  |  |  |
| Surgery only | 1.00 |  | <.001 | 1.00 |  | <.001 |
| Surgery & Chemo or radiotherapy | 1.89 | (1.83–1.94) |  | 1.50 | (1.45–1.54) |  |
| Chemo or radiotherapy only | 1.51 | (1.39–1.63) |  | 1.48 | (1.35–1.61) |  |
| **Death** |  |  |  |  |  |  |
| Survivor | 1.00 |  | <.001 | 1.00 |  | <.001 |
| Died | 2.52 | (2.38–2.66) |  | 3.92 | (3.78–4.07) |  |
| **Year of colorectal cancer incidence** | 1.00 | (0.99–1.01) | 0.82 | 1.02 | (1.01–1.02) | <.001 |
| *A regression analysis using GEE model with gamma distribution and log-link function*  *Note: NHI: National Health Insurance; RR: Relative risk; CI: confidence interval; CCI: Charlson comorbidity index; LOS: Length of stay* | | | | | | |
